# Analysis of Expression and Functional Activity of Aromatic L-Amino Acid Decarboxylase (DDC) and Serotonin Transporter (SERT) as Potential Sources of Serotonin in Mouse Ovary

**DOI:** 10.3390/ijms20123070

**Published:** 2019-06-23

**Authors:** Denis A. Nikishin, Nina M. Alyoshina, Maria L. Semenova, Yuri B. Shmukler

**Affiliations:** 1 N.K. Koltzov Institute of Developmental Biology, Russian Academy of Sciences, Vavilova Street, 26, Moscow 119334, Russia; ninalyoshina@gmail.com (N.M.A.); yurishmukler@yahoo.com (Y.B.S.); 2 Faculty of Biology, Lomonosov Moscow State University, Leninskie Gory, 1, bld. 12, Moscow 119991, Russia; mlsemenova@gmail.com

**Keywords:** mouse, ovary, serotonin, SERT, DDC, fluoxetine

## Abstract

The origin of serotonin in the ovary is the key question for understanding mechanisms of serotonergic regulation of reproductive function. We performed a study of the expression and functional activity of the serotonin transporter (SERT) and the enzyme for the synthesis of serotonin, aromatic l-amino acid decarboxylase (DDC) in mouse ovary. A pronounced peak of SERT mRNA expression occurs at the age of 14 days, but serotonin synthesis enzymes are expressed at the maximum level in the ovaries of newborn mice. SERT is detected immunohistochemically in all cellular compartments of the ovary with a maximum level of immunostaining in the oocytes of growing ovarian follicles. DDC immunolocalization, in contrast, is detected to a greater extent in primordial follicle oocytes, and decreases at the later stages of folliculogenesis. Serotonin synthesis in all cellular compartments occurs at very low levels, whereas specific serotonin uptake is clearly present, leading to a significant increase in serotonin content in the oocytes of growing primary and secondary follicles. These data indicate that the main mechanism of serotonin accumulation in mouse ovary is its uptake by the specific SERT membrane transporter, which is active in the oocytes of the growing ovarian follicles.

## 1. Introduction

Serotonin (5-hydroxytryptamine, 5HT) is found in the reproductive system of female mammals, including the ovaries [1], follicular fluid [2], mature oocytes and cumulus cells [3]. One of the conservative functions of serotonin is the modulation of egg maturation in a wide variety of animal groups, including mammals [4,5,6,7]. The concentration of serotonin in the ovary changes during the reproductive cycle [1] and correlates with certain pathological processes [2], as well as with the success of the in vitro fertilization procedure [8]. In addition, serotonin affects the functional activity of follicular cells, which not only play an important role in the process of maturation of the egg but are also the main source of oestrogen in the female body [5,6,9,10].

The origin of serotonin in the ovary is the key question for understanding mechanisms of serotonergic regulation of reproductive function. The enzyme in the first step of serotonin biosynthesis, tryptophan hydroxylase, *TPH1*, is expressed in cumulus cells [3] and *TPH2* is expressed in mature oocytes [11]. Because the tryptophan hydroxylase is a rate-limiting enzyme, it is believed that a local system of serotonin synthesis is present in the ovary. However, it is worth noting that the synthesis of serotonin by a second enzyme, aromatic L-amino acid decarboxylase, DDC, does not occur directly in the ovaries of mammals. Earlier, we showed that *DDC* is expressed at very low levels in granulosa cells and mature oocytes. Platelets of the bloodstream, mast cells localized in the stroma of the ovary and the few nerve fibres that accompany the large medullary vessels, are potential sources of serotonin that is exogenous to the follicle [12]. The expression and the activity of the serotonin membrane transporter SERT are shown both in cumulus cells and in isolated oocytes [3].

It appears that serotonin uptake is present throughout ovaries, whereas serotonin synthesis in the ovary is a less significant mechanism. However, there are no data on the temporal characteristics of synthesis and membrane transport in the growing ovarian follicles. Given the potential role of serotonin as a regulator of the process of folliculogenesis, the identification of the synthesis and membrane transport of serotonin in the developing ovarian follicle remains a fundamental issue. To clarify the role of serotonin synthesis and uptake in the regulation of ovarian function, a study on the dynamics of expression, localization and functional activity of the key factors–the serotonin synthesis enzyme DDC and the serotonin transporter SERT–was carried out.

## 2. Results

### 2.1. Gene Expression Profiles of the Serotonin Transporter SERT and Enzymes of Serotonin Synthesis DDC, TPH1 and TPH2, during Postnatal Development of Mouse Ovary

**Figure 1:**
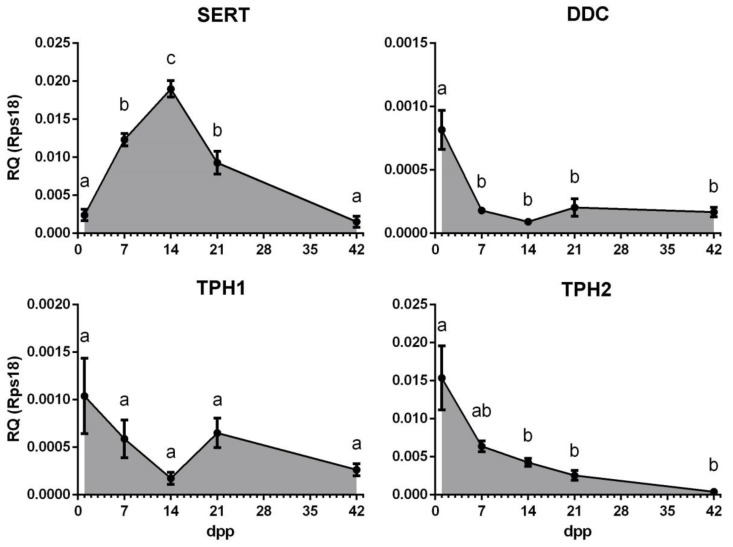
Gene expression profiles of the serotonin transporter *SERT* and enzymes of serotonin synthesis *DDC*, *TPH1* and *TPH2*, during postnatal development of the mouse ovary. The relative quantity (RQ) was calculated using 2^−ΔCt^ method relative to the reference gene *RPS18* (M ± SEM). Different letters denote statistical significance between groups at *p* < 0.05, according to ordinary one-way ANOVA with Holm-Sidak’s multiple comparisons test.

During the postnatal period in female mice, a gradual activation of the growth of ovarian follicles occurs, and more and more progressive stages of folliculogenesis consistently appear in the ovary. We performed a quantitative study of the gene expression of components presumably responsible for the synthesis and uptake of serotonin in postnatal mouse ovaries in order to identify the dynamics of their expression and draw conclusions about the period of folliculogenesis during which these mechanisms may be active (Figure 1). The age-related dynamics of the *SERT* gene expression show a pronounced peak at the age of 14 days postpartum (dpp), when growing follicles predominate in the ovary, and a significantly lower level of expression at earlier and later stages of development. The expression of the *DDC* gene is maximal in the ovaries of newborn mice, when the vast majority of the follicles are in the primordial stage, and it decreases slightly in the later stages. Expression levels of the *TPH1* and *TPH2* genes also have a maximum in the ovaries of newborn mice and then decrease thereafter.

Based on the results obtained, we assumed that serotonin transport is maximally active during the period of follicle growth. At the same time, the activity of the synthesis system is most likely to be confined to earlier stages of oogenesis.

### 2.2. Localization of SERT and DDC Immunoreactivity in Mouse Ovary

We performed an immunohistochemical study to establish the localization of the serotonin transporter SERT and aromatic L-amino acid decarboxylase DDC in the mouse ovary. The study was performed on both prepubertal (14 dpp) and adult mice.

**Figure 2:**
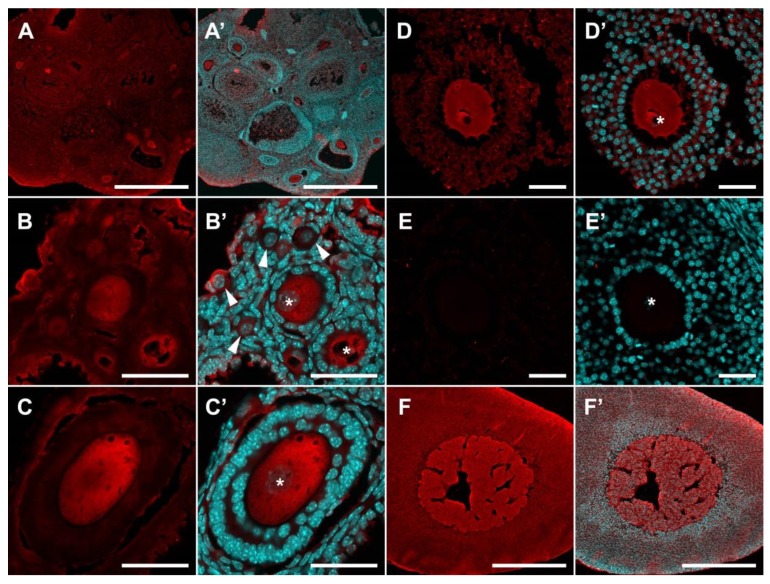
Immunohistochemical detection of SERT in the mouse ovary. (**A**–**D**) SERT immunoreactivity in the ovary of prepubertal (**B**,**C**) and adult mice (**A**,**D**). (**E**) Negative control: Ovary immunostaining without primary antibodies. (**F**) Positive control: Adrenal gland with SERT immunoreactivity in the medullar region. (**A’**–**F’**) Images merged with nuclear staining. Twenty-μm cryosections were stained for SERT (red false colour) and nuclei were dyed with DAPI counterstain (cyan false colour). Arrowheads point to primordial follicles. Asterisks mark oocytes in growing follicles. Scale bars: (**A**,**F**) 500 µm; (**B**–**E**) 50 µm.

SERT immunoreactivity is detected in all cellular compartments of the ovary, including ovarian follicles, both in oocytes and in follicular cells (Figure 2). However, while the intensity of immunostaining in primordial follicles is low, it becomes noticeably more pronounced in primary single-layer follicles (Figure 2B). The most intensive immunostaining is observed in oocytes of pre-antral and antral follicles (Figure 2C,D). Immunostaining is observed throughout the cytoplasm but is much less intense in the region of the nucleus. In the areas of the greatest intensity, immunostaining acquires a fine, granular structure. We performed a quantitative analysis of the intensity of immunofluorescence in individual cellular compartments of the ovary (Figure 4B). SERT immunoreactivity in granulosa cells of primordial follicles and granulosa and theca cells of growing follicles, is at the same low level. Immunofluorescence is much higher in the oocytes of growing follicles (by approximately 2.3 times) and is at an intermediate level in oocytes of primordial follicles. It is important to note that the differences between different groups of oocytes are statistically significant.

**Figure 3:**
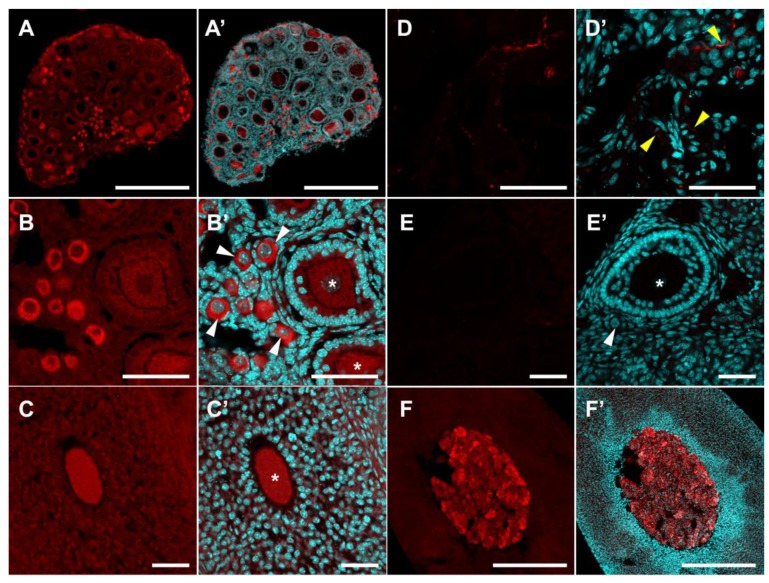
Immunohistochemical detection of DDC in the mouse ovary. (**A**–**D**) DDC immunoreactivity in the ovary of prepubertal (**A**,**B**,**D**) and adult mice (**C**). (**E**) Negative control: Ovary immunostaining without primary antibodies. (**F**) Positive control: Adrenal gland with DDC immunoreactivity in the medullar region. (**A’**–**F’**) Images merged with nuclear staining. Twenty-μm cryosections were stained for DDC (red false colour) and nuclei were dyed with DAPI counterstain (cyan false colour). White arrowheads point to primordial follicles. Asterisks mark oocytes in growing follicles. Yellow arrowheads point to nerve fibres in ovarian stromal tissue. Scale bars: (**A**,**F**) 500 µm; (**B**–**E**) 50 µm.

**Figure 4:**
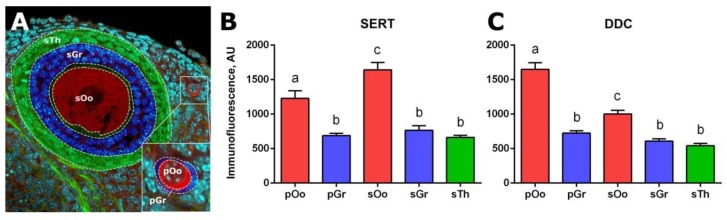
Quantitative analysis of anti-SERT and anti-DDC immunoreactivity in individual cellular compartments of mouse ovaries. (**A**) Cell compartments of the ovary. Dashed lines mark the areas to be analysed: sOo–oocyte, sGr–granulosa cells, sTh–theca cells of the secondary (preantral) follicle; pOo–oocyte, pGr–granulosa cells of the primordial follicle. (**B**) Quantitative analysis of SERT immunoreactivity. (**C**) Quantitative analysis of DDC immunoreactivity. AU–arbitrary units of immunofluorescence. Different letters denote statistical significance between groups at *p* < 0.05, according to ordinary one-way ANOVA with Holm-Sidak’s multiple comparisons test.

An immunohistochemical study of DDC localization revealed that a slight background level of immunostaining was observed in granulosa cells, theca cells and ovarian stroma. The DDC immunoreactivity in the fibres of adrenergic neurons innervating the ovary (Figure 3D), as well as in mast cells, is detected in the stromal tissue. In addition, intense immunostaining is detected in oocytes of primordial follicles (Figure 3A,B). At the later stages of folliculogenesis, starting with the primary single-layer follicles, the intensity of immunostaining gradually decreases to the baseline level (Figure 3B,C). Immunostaining of decarboxylase in oocytes has a fine, granular structure and is detected throughout the cytoplasm but does not extend to the region of the nucleus. We performed a quantitative analysis of DDC immunoreactivity in ovarian cellular compartments. DDC immunoreactivity is low in granulosa cells of primordial follicles and in granulosa and theca cells of growing follicles. Immunofluorescence in the oocytes of primordial follicles is approximately 2.6 times higher. DDC immunoreactivity is at an intermediate level in oocytes of growing follicles. The differences between different groups of oocytes are statistically significant (Figure 4C).

Similar data were obtained by staining the ovaries of 14-day-old and adult mice (Figure 2A,D and Figure 3C). This suggests that the dynamics observed in Figure 1 are primarily associated with changes in the morphology and proportion of follicles and stromal structures in the ovary. Negative control stainings without first antibodies are shown in Figure 2E and Figure 3E. We also performed adrenal gland immunostaining as a positive control for antibody reactions, and both SERT and DDC were strongly detected in the adrenal medulla (Figure 2F and Figure 3F).

### 2.3. Functional Activity of Serotonin Synthesis and Uptake in Mouse Ovary

We performed experiments on the short-term incubation of ovarian fragments with modulators of serotonin concentration to detect the activity of serotonin synthesis and uptake systems. An age of 14 dpp was chosen for the study, since, at this time, a large number of both growing and primordial follicles can be found in the mouse ovary (see Figure 3A). Moreover, a decrease in the expression level of both genes is observed at later stages of ovary development.

**Figure 5:**
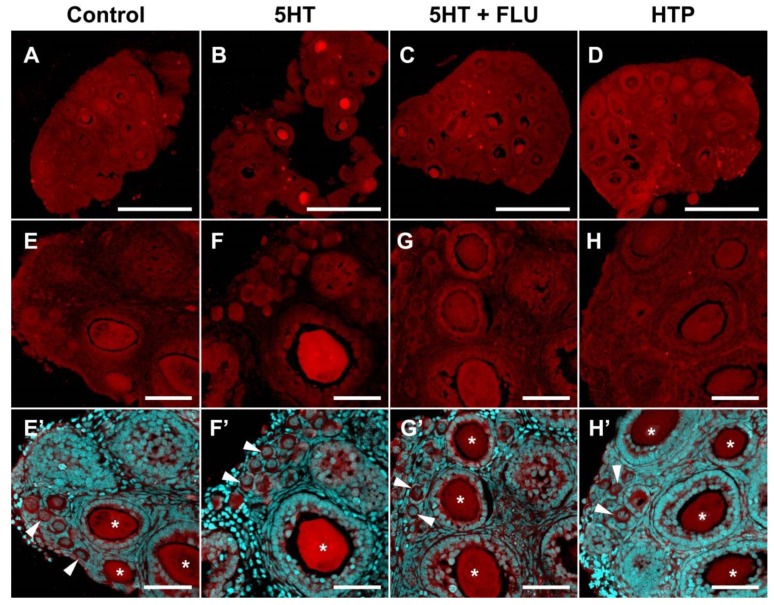
Functional activity of uptake and synthesis of serotonin in the prepubertal (14 dpp) mouse ovary. (**A**,**E**) Control. (**B**,**F**) Two-h incubation with serotonin (5HT) (1 μM). (**C**,**G**) Two-h incubation with 5HT (1 μM) with previous addition of fluoxetine (FLU) (10 μM). (**D**,**H**) Two-h incubation with 5-hydroxytryptophan HTP (10 μM). (**E’**–**H’**) Images merged with nuclear staining. Twenty-μm cryosections were stained for serotonin (red false colour) and nuclei were dyed with DAPI counterstain (cyan false colour). White arrowheads point to primordial follicles. Asterisks mark oocytes in growing follicles. Scale bars: (**A**–**D**) 500 µm; (**E**–**H**) 50 µm.

**Figure 6:**
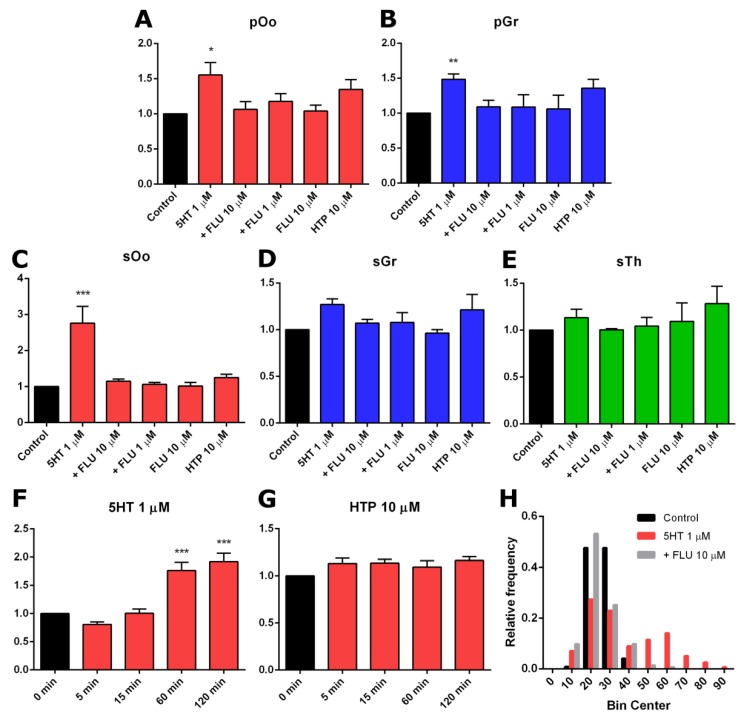
Quantitative analysis of serotonin accumulation in the study of the activity of the systems of its synthesis and uptake in mouse ovaries. (**A**–**E**) Quantitative analysis of anti-serotonin immunoreactivity in individual cellular compartments of mouse ovaries after incubation with serotonin (5HT), with 5HT and previous addition of fluoxetine (+FLU), with fluoxetine only (FLU) and with 5-hydroxytryptophan (HTP): sOo–oocyte, sGr–granulosa cells and sTh–theca cells of the secondary (preantral) follicle; pOo–oocyte and pGr–granulosa cells of primordial follicle (M ± SEM). The level of immunofluorescence in the control sample is taken as 1. * Denotes statistical significance at *p* < 0.05, ** at *p* < 0.01 and *** at *p* < 0.005, between control and treated groups, using the Friedman test. (**F**,**G**) The temporal dynamics of serotonin accumulation in oocytes of growing follicles (sOo) during incubation with 5HT (1 μM) and HTP (10 μM) (M ± SEM). *** Denotes statistical significance at *p* < 0.005, between control and treated groups, using the Friedman test. (**H**) Frequency distribution histogram of serotonin accumulation in oocytes of growing follicles (sOo). A bimodal distribution is observed in the 5HT group.

An increase in immunofluorescence intensity was observed in fragments of the ovary incubated in 1 μM serotonin for 2 h (Figure 5B,F). This effect is most pronounced in large oocytes of growing ovarian follicles. We performed a quantitative calculation of serotonin accumulation for various cellular compartments of the developing ovarian follicle. In primordial follicles, an increase in the levels of immunofluorescence is observed in oocytes (Figure 6A) and follicular cells (Figure 6B). The differences from the control in both cases are significant. In growing ovarian follicles, an increase in the level of 5HT immunofluorescence occurs in oocytes (Figure 6C), as well as in granulosa and theca cells (Figure 6D,E). In the case of oocytes, the differences are statistically significant. Thus, the functional activity of serotonin uptake is detected in the oocytes of growing follicles and, to a lesser extent, in primordial follicles.

We performed similar experiments with preliminary addition of the selective serotonin reuptake inhibitor fluoxetine, in order to show the specificity of the effects. The addition of fluoxetine (10 μM and 1 μM) to ovarian fragments resulted in the absence of a significant accumulation of serotonin (Figure 5C,G and Figure 6A–E). Adding fluoxetine alone does not affect the serotonin content in the ovary (Figure 6A–E). These results suggest that the serotonin uptake observed in the ovary is specific and is carried out by SERT.

We investigated the activity of the serotonin synthesis system in the ovary. Addition of the serotonin biochemical precursor 5-hydroxytryptophan (10 μM) to ovarian fragments leads to a slight increase in immunofluorescence levels (Figure 6A–E), but the effect is not statistically significant. Although DDC is expressed in the ovary, it appeared to be in an inactive state during the study period.

A study of the temporal dynamics of the accumulation of serotonin in the oocytes of secondary ovarian follicles after the addition of serotonin and 5-hydroxytryptophan, was carried out. It was established that significant differences in the serotonin content appear 1 h after the start of incubation with serotonin (Figure 6F), whereas the accumulation of serotonin with the addition of 5-hydroxytryptophan (10 µM, 2 h) does not occur (Figure 6G).

## 3. Discussion

In this study, we investigated the expression and functional activity of the two most likely mechanisms of serotonin accumulation in the mouse ovary, i.e., synthesis via DDC and uptake using SERT. We found that mRNA enzymes for serotonin synthesis are expressed at a low level in the ovary. At the same time, DDC is detected immunohistochemically, along with components of the ovarian stroma, in oocytes of primordial follicles. However, pronounced activity of this enzyme is not detected in the components of ovarian follicles. *SERT*-gene mRNA is expressed at a fairly high level and shows a peak of expression at the age of 14 dpp, when growing ovarian follicles dominate in the ovary. Its immunoreactivity and specific activity are detected at a high level in the oocytes of the growing follicles.

The first step of serotonin biosynthesis, hydroxylation, is rate-limiting [13]. Therefore, the presence of TPH is often erroneously considered as a sufficient condition for the synthesis of serotonin in tissue [14]. The second synthesis enzyme, aromatic L-amino acid decarboxylase, DDC, is often accepted as ubiquitous and constantly expressed. However, it is not expressed in all cell types and is often the missing link in the serotonin synthesis system [15]. According to the results of quantitative PCR studies, serotonin synthesis enzymes are expressed in the ovary at a relatively low level (Figure 1). Oocyte-specific *TPH2* expression [11] is most pronounced in the mouse ovary, while *TPH1* and *DDC* have significantly lower expression levels. Considering that the presence of DDC is a necessary condition for the synthesis of serotonin and that 5-hydroxytryptophan can have exogenous sources such as the bloodstream [16], it was important for us to first study the expression and activity of DDC in the mouse ovary. All the serotonin synthesis enzymes show a tendency for their expression levels to decrease during postnatal development (Figure 1). The maximum expression level of *DDC* is observed in the newborn mouse ovaries, in which the formation of a primordial follicular pool comes to completion [17]. The *DDC* expression in the ovary reaches a minimum value by the age of 14 dpp when the first wave of folliculogenesis occurs. An immunohistochemical study of the localization of the enzyme in the ovary confirmed this result: DDC is detected in oocytes of primordial follicles, whereas at later stages of folliculogenesis its immunoreactivity is significantly reduced (Figure 4). However, the analysis of decarboxylase activity in experiments on the incubation of ovarian fragments with the biochemical precursor of serotonin, did not reveal pronounced enzyme activity in primordial or growing ovarian follicles (Figure 4 and Figure 5). The concentration of HTP used in the experiments was several times higher than the physiological concentration in plasma [16]. Apparently, despite the identified expression of DDC, it is inactive in the ovarian follicles. According to our findings, we can conclude that synthesis cannot be a significant source of serotonin in the oocyte and ovarian follicle in the postnatal ovary. Moreover, the lack of decarboxylase activity in the components of the ovarian follicles also indicates the inactivity of the synthesis of dopamine, another important product of this enzyme. It is possible that DDC activity may take place and may play a certain role at earlier stages of ovarian development, and that expression of the enzyme in primordial follicles is residual. However, there are no specific data that would indicate this yet. In addition, decarboxylase activities in mast cells and nerve fibres in the stroma of the ovary can certainly be present. However, the analysis of the values of these elements is beyond the scope of this work.

A very probable mechanism for the accumulation of serotonin in oocytes is specific membrane transport by SERT. A quantitative real-time PCR study showed that *SERT* is expressed at a fairly high level in the ovary (Figure 1). The age dynamics of its expression show a pronounced peak, occurring at 14 dpp. This indicates its activity during the period of follicular growth. The first wave of active folliculogenesis occurs in mice of this age. A number of growing follicles are observed in the ovary, but they do not reach maturation and ovulation for physiological reasons [18]. It was shown that the follicles and oocytes activated during this period are viable and capable of maturation in vitro [19]. An immunohistochemical study of the localization of the transporter in the ovary confirmed that the most pronounced expression of SERT is observed in the oocytes of growing ovarian follicles (Figure 2). Moreover, an experimental study showed that the serotonin uptake system is active in the oocytes of the growing ovarian follicles. Serotonin uptake is also reliably detected in oocytes and follicular cells of primordial follicles, although to a lesser extent. The serotonin concentration used in the experiments is approximately equal to its concentration in follicular fluid [2]. 

An important issue is the specificity of the observed accumulation of serotonin in the oocytes of the growing follicles. In addition to SERT, a number of other mechanisms can lead to the accumulation of serotonin in the cell, for example (at high concentrations of serotonin), the dopamine transporter (DAT) and the norepinephrine transporter (NET), as well as poly-specific transporters, such as organic cation transporters (OCT) and the plasma membrane monoamine transporter (PMAT) [20]. The previous addition of a selective serotonin reuptake inhibitor, fluoxetine, completely prevents the accumulation effect that confirms the involvement of the specific SERT transporter in this process. We used a fluoxetine concentration of 10 μM, which was shown to be effective in experiments on mouse embryos pre-implantation [21]. However, in our experiments, fluoxetine also acted effectively at a concentration of 1 μM (Figure 6). Fluoxetine has some affinity for DAT and NET, although it is orders of magnitude lower than that for SERT [22]. Both transporters are expressed in the ovary in granulosa cells [23]. However, there was no accumulation of serotonin in the experiment, which indicates that these mechanisms do not contribute to the observed effect. As for nonspecific transporters, it is shown that a concentration of fluoxetine of 1 μM is ineffective as a blockade [24]. However, the presence of a detectable serotonin concentration in cells that do not exhibit active uptake, leaves open the question of the source of serotonin in these cases. In all likelihood, there are some nonspecific mechanisms operating with low efficiency. Nevertheless, the uptake of serotonin into the oocytes by SERT is active during the period of follicular growth and can ensure its accumulation.

It is interesting that a bimodal distribution describes the activity of serotonin uptake in the oocytes of growing follicles. It seems that the oocytes fall into two groups, according to their ability to accumulate serotonin: Those in which the seizure is very active and those in which it is practically inactive. Such a distribution is observed in both 14-dpp and adult ovaries and is not coupled with the size of the follicle and oocyte. The reasons for these differences are not yet clear. Morphologically, these follicles do not differ. The probable reason is differences in the functional status of oocytes, which may determine the process of selection of oocytes during maturation. So far, there are no suggestions about whether active serotonin uptake tends to mark potential oocytes that can complete oogenesis or less successful follicles that go into atresia. In addition, the difference in serotonin content may also be associated with different levels of metabolism—through degradation enzymes or melatonin synthesis enzymes. This question requires further investigation, including the use of direct methods for the quantitative measurement of monoamines in tissue. We should separately note that the study of the activity of the serotonergic system, based on measuring the level of immunofluorescence, is an indirect method and is not quantitative in the full sense of the word. However, this approach has its advantages over direct methods, as it allows the effects in different cellular compartments to be easily differentiated, without using cell dissociation and leaving the experimental tissue in an almost native state.

The further fate of serotonin accumulated in the oocyte is unknown. According to the classical transmitter mechanism, serotonin can accumulate in vesicular structures and subsequently perform an intercellular signalling function locally around the oocyte or in early embryos. The expression of the vesicular transporter of monoamines, *VMAT2*, which accumulates serotonin into acid vesicles in oocytes and early embryos, is shown in our earlier work [15]. In addition, the fine-grained pattern of serotonin immunolocalization often observed in oocytes, both in the experiment and the control, supports this suggestion. Given the activity of this mechanism during follicular growth, we assume that serotonin will affect the processes of the final stages of oogenesis, i.e., the completion of meiosis, and the functional state of the oocyte, which can also affect the functional activity of follicular cells. In hamsters and rats, serotonin specifically stimulates the synthesis of oestradiol in pre-ovulatory follicles cultured in vitro [5,6]. Stimulation of steroidogenesis by serotonin has also been observed in human granulosa cell cultures [9,10]. The mechanism of serotonin-dependent regulation of follicular cells’ functional activity remains unclear, but it is known that uptake by membrane transporter SERT plays a key role in this process. In SERT knockout mice, aromatase expression is depressed and, as a result, blood oestradiol levels decrease. A similar effect is caused by the selective serotonin reuptake inhibitor (SSRI) paroxetine [25]. Similar results were obtained in other model species, for example, in the bony fish *Danio rerio*, where SSRI fluoxetine leads to a decrease in the number of eggs, as well as in the expression level of aromatase mRNA and the ovarian oestradiol content [26].

On the other hand, serotonin may exhibit intracellular activity, having accumulated in the oocyte. The biochemical role of serotonin as a substrate for the synthesis of another important hormone, melatonin [27], also cannot be excluded. There is evidence of the presence of the expression of melatonin synthesis enzymes in oocytes, follicular cells and early embryos [28]. Additionally, the mechanisms of post-translational modification of proteins by covalently attaching serotonin (serotonylation), are known. These mechanisms are active during early development of invertebrates and have long-term consequences on their behaviour [29].

Summarizing the above, we can conclude that the main source for the accumulation of serotonin in the mouse ovary is the mechanism of serotonin uptake through a specific SERT membrane transporter, which is active in the oocytes of the growing ovarian follicles. Due to the lack of serotonin synthesis during this period, it turns out that serotonin accumulated in mouse oocytes is almost completely maternal. The direct source of serotonin in the ovary is most likely to be blood vessels, as well as mast cells [12]. Considering that a full-fledged serotonergic signalling system functions in the ovary [14], it is important to note that accumulated serotonin may play an important role in regulating the physiological function of the ovary and in pathological processes. Several serotonin receptors are expressed in oocytes and follicular cells that are capable of activating various systems of secondary messengers [15]. Among these, inositols are particularly worth noting. These play an important role in regulating the maturation of oocytes [30], affect embryo quality in patients undergoing in vitro fertilization procedure [31] and are involved in the pathogenesis of ovarian diseases [32].

Thus, the mechanism of accumulation of maternal serotonin in oocytes at these stages can provide a link between the physiological state of the female and some functional parameters of the processes of oocyte maturation and subsequent embryonic development. It is important to note that the serotonin uptake mechanism is ubiquitous in the early development of most animals, which indicates its fundamental importance [26,33]. This fact is extremely important for humans, due to the wide distribution of neuroactive drugs in the treatment of a huge number of diseases, including the wide distribution and uncontrolled use of SSRI antidepressants [34]. The use of such substances will inevitably affect the accumulation of serotonin during the period of follicular growth and can cause long-term health effects [21,35,36,37]. On the other hand, there is a large quantity of data on the effects of lifestyle and stress on female fertility [38,39]. In this regard, the modulation of serotonin using pharmacological substances such as SSRIs, can potentially affect women’s reproductive function [40]. At any rate, the study of the serotonergic regulation of female reproductive function awaits continuation.

## 4. Materials and Methods

### 4.1. Experimental Animals

C57BL/6 female mice obtained from the Laboratory Animal Centre of Koltzov Institute of Developmental Biology, RAS, were used in animal experiments. Animals were maintained under controlled conditions (22–24 °C and 14L:10D photoperiod). Mice were given ad libitum access to food and water. Experiments were carried out in accordance with the European Communities Council Directive of 24 November 1986 (86/609/EEC). All the protocols of manipulations with animals have been approved by the Commission on Bioethics of Koltzov Institute of Developmental Biology, Russian Academy of Sciences (Project identification code: №23, Date: 15 November 2018).

### 4.2. Quantitative Polymerase Chain Reaction (qPCR)

Ovaries were isolated from mice at the age of 1 day postpartum (dpp), 7 dpp, 14 dpp, 21 dpp, and 42 dpp for a quantitative study of gene expression. Total RNA was isolated using RNA extraction reagent (Evrogen, Moscow, Russia). Next, DNase I (Thermo Fisher Scientific, Waltham, MA, USA) treatment was carried out, and 1 μg of the total RNA from each group was reverse transcribed using random hexadeoxynucleotides and Moloney murine leukaemia virus reverse transcriptase (Evrogen, Moscow, Russia), according to the manufacturer’s instructions. Quantitative real-time PCR was conducted to quantify mRNA levels of target genes by using the StepOnePlus Real-Time PCR System (Thermo Fisher Scientific, Waltham, MA, USA) and a qPCRmix-HS SYBR + HighROX kit (Evrogen, Moscow, Russia). In each experiment, the expression levels of the selected genes in every group were normalized to the reference gene using the 2^−ΔCt^ method, under the same threshold condition for all genes. Ribosomal protein gene *RPS18* was used as a reference gene because is stably expressed in ovarian tissue [41]. Analysis of the melting curve and examination of the correct melting point of the PCR product (Table 1) were used to assess the specificity of the reaction. All primer sequences used for performing qPCR are listed in Table 1.

### 4.3. In Vitro Ovary Incubation Experiments

Mouse ovaries at 14 dpp were isolated, dissected into eight fragments and cultured in four-well culture dishes (Nunc, Roskilde, Denmark) containing 1 mL DMEM/F-12 medium (Gibco, Gaithersburg, MD, USA) supplemented with chemicals at 37 °C in 5% CO_2_. The chemicals used in the study were serotonin creatinine sulfate (1 μM; H7752 Sigma-Aldrich, St. Louis, MO, USA), fluoxetine hydrochloride (10 μM; PHR1394 Sigma-Aldrich, St. Louis, MO, USA) and 5-hydroxy-L-tryptophan (10 μM; H9772 Sigma-Aldrich, St. Louis, MO, USA). The ovaries from three to five animals were used for each experiment.

### 4.4. Immunohistochemistry

For immunofluorescence, ovaries or their fragments were fixed in 4% paraformaldehyde (PFA) overnight at 4 °C, followed by incubation for 1 h in 15% (*w*/*v*) sucrose and overnight in 30% (*w*/*v*) sucrose, followed by embedding in Tissue-Tek^®^ O.C.T.™ Compound (Sakura Finetek Japan Co., Ltd., Chiba, Japan). The specimens were stored at −80 °C before cutting. Twenty-μm cryosections of all comparison groups were mounted on the same glass slide to avoid differences in the conditions of subsequent immunostaining. Slides were then washed in phosphate-buffered saline (PBS) and permeabilized in 0.1% (*v*/*v*) Triton X-100 for 15 min. For DDC staining, antigen retrieval was performed by placing slides in 1% sodium dodecyl sulfate solution for 5 min. and washing in PBS. Sections were blocked with 5% (*w*/*v*) bovine serum albumin (BSA), followed by sequential incubations with primary and secondary antibodies (Table 2). The specificity of all primary antibodies was examined by omitting primary antibodies in immunostaining. DNA was counterstained with 4′,6-diamidino-2-phenylindole (Sigma-Aldrich, St. Louis, MO, USA). Samples were mounted in Fluoroshield Mounting Medium (Abcam, Cambridge, UK).

### 4.5. Estimation of Serotonin Immunoreactivity

Digital images of the middle optical section in 10–20 randomized slices of each group to be analysed were obtained using a laser scanning confocal microscope FVi10 (Olympus, Tokyo, Japan). All image parameters, including pinhole size, detector gain, amplifier offset, amplifier gain and laser intensity, were first set using the 5HT group, and the same setting was then used for all samples imaged. Frame size, scan speed and averaging were the same for all images. Images were converted to a 16-bit greyscale format and analysed using ImageJ software. The analysed cell compartments (Figure 4A) were selected as the region of interest (ROI) in the DAPI channel and then the immunofluorescence intensity was measured in the immunostaining channel. The immunofluorescence intensity in arbitrary units (AU) is the average intensity of all pixels (mean grey value) present in the ROI. When analyzing the experimental samples, normalization for immunofluorescence in the control sample (taken as 1) was performed. To minimize bias, the examiners did not know the identities of the sections. The data were then analysed for statistical differences with GraphPad Prism 5.0 (GraphPad Software, Inc., San Diego, CA, USA), using ordinary one-way ANOVA with Holm-Sidak’s multiple comparisons test or the Friedman test.

## 5. Conclusions

The analysis of the expression and functional activity of the serotonin transporter (SERT) and the enzyme for the synthesis of serotonin, aromatic L-amino acid decarboxylase (DDC) as the main possible mechanisms of serotonin accumulation in mouse ovary is carried out. Received data indicate that the main source for the accumulation of serotonin in the mouse ovary is its uptake by the specific SERT membrane transporter, which is active in the oocytes of the growing ovarian follicles.

## Figures and Tables

**Table 1:** Oligonucleotide primers used for quantitative real-time PCR (qPCR).

Gene Name	NCBI Gene ID	Forward Primer	Reverse Primer	PCR Product Length, bp	PCR Product Tm, °C
*SERT (SLC6A4)*	15567	GGGAGACCTGGGGCAAGAAG	CAGGGCGAGCTCCATGTAGAAGA	182	87.1
*DDC*	13195	TCCCCACGGCTAGCTCATACCC	TTCCCCAGCCAGTCCATCATCA	133	86.5
*TPH1*	21990	TGCGACATCAGCCGAGAACAGT	GGCGCAGAAGTCCAGGTCAGA	162	86.4
*TPH2*	216343	CATGGCTCCGACCCCCTCTACA	ATACGCCCGCAGTTGACCCTCTT	219	86.8
*RPS18*	20084	AAGAAAATTCGAGCCCATAGAGG	TAACAGCAAAGGCCCAGAGACT	138	86.1

**Table 2:** Antibodies used for immunohistochemistry.

Antibody	Manufacturer	Catalogue Number	Dilution
Goat polyclonal Anti-Serotonin transporter (Sert) antibody	Abcam, UK	ab130130	1:500
Rabbit polyclonal Anti-DOPA Decarboxylase (Ddc) antibody	Abcam, UK	ab3905	1:500
Anti-Serotonin antibody produced in rabbitwhole antiserum	Sigma-Aldrich, Germany	S5545	1:1000
Fluorescein (FITC) AffiniPure Goat Anti-Rabbit IgG (H + L)	Jackson Immuno Research, UK	111-095-003	1:200
Donkey F(ab’)2 Anti-Rabbit IgG H&L (Alexa Fluor^®^ 568) preadsorbed	Abcam, UK	ab175694	1:200
Donkey F(ab’)2 Anti-Goat IgG H&L (Alexa Fluor^®^ 647) preadsorbed	Abcam, UK	ab150139	1:200

## Abbreviations

AU	arbitrary units
BSA	bovine serum albumin
dpp	day postpartum
5HT	5-hydroxytryptamine, serotonin
HTP	5-hydroxy-L-tryptophan
PBS	phosphate buffered saline
PCR	polymerase chain reaction
PFA	paraformaldehyde
qPCR	quantitative real-time PCR
ROI	region of interest
SSRI	selective serotonin reuptake inhibitor
